# A Cross-Layer Duty Cycle MAC Protocol Supporting a Pipeline Feature for Wireless Sensor Networks

**DOI:** 10.3390/s110505183

**Published:** 2011-05-11

**Authors:** Fei Tong, Rong Xie, Lei Shu, Young-Chon Kim

**Affiliations:** 1 Department of Computer Engineering, Chonbuk National University, Jeonju 561-756, Korea; E-Mails: tong1987fei@163.com (F.T.); xierong@jbnu.ac.kr (R.X.); 2 Department of Multimedia Engineering, Osaka University, Osaka 565-0871, Japan; E-Mail: lei.shu@ieee.org

**Keywords:** MAC protocol, cross-layer, duty cycle, pipeline feature, wireless sensor networks

## Abstract

Although the conventional duty cycle MAC protocols for Wireless Sensor Networks (WSNs) such as RMAC perform well in terms of saving energy and reducing end-to-end delivery latency, they were designed independently and require an extra routing protocol in the network layer to provide path information for the MAC layer. In this paper, we propose a new cross-layer duty cycle MAC protocol with data forwarding supporting a pipeline feature (P-MAC) for WSNs. P-MAC first divides the whole network into many grades around the sink. Each node identifies its grade according to its logical hop distance to the sink and simultaneously establishes a sleep/wakeup schedule using the grade information. Those nodes in the same grade keep the same schedule, which is staggered with the schedule of the nodes in the adjacent grade. Then a variation of the RTS/CTS handshake mechanism is used to forward data continuously in a pipeline fashion from the higher grade to the lower grade nodes and finally to the sink. No extra routing overhead is needed, thus increasing the network scalability while maintaining the superiority of duty-cycling. The simulation results in OPNET show that P-MAC has better performance than S-MAC and RMAC in terms of packet delivery latency and energy efficiency.

## 1. Introduction

The limitation of energy due to the limited battery capacity of sensor nodes is a fundamental problem in Wireless Sensor Networks (WSNs). Communication protocols for WSNs, including routing and MAC layer protocols should thus be designed energy-efficiently. Traditional wireless MAC protocols such as IEEE 802.11 are not suitable for this purpose since in these protocols nodes are required to stay awake to listen to the medium, even when the network becomes idle. This inefficient idle-listening mechanism wastes substantial energy [1,2].

Nowadays, many methods introduce duty-cycling mechanisms into MAC designs for WSNs to achieve low energy consumption. In the duty-cycling approach, each node periodically experiences an active state and a sleeping state. When in the active state, a node listens to the radio channel for possible transmissions, whereas in the sleeping state, it turns off its radio to save energy. Each node establishes and maintains a schedule to indicate when it should wake up or sleep based on the synchronization requirements among neighboring nodes.

S-MAC [3] is a typical synchronized duty cycle MAC protocol for WSNs. In S-MAC, each node maintains a fixed listening/sleeping schedule. The listening interval is divided into two parts, namely *SYNC* and *DATA*. The *SYNC* part is for synchronization among neighboring nodes using SYNC packets, and the *DATA* part is for data transmission using the RTS/CTS handshake mechanism as in 802.11. Although S-MAC is energy efficient, it may introduce significant packet delivery latency, since a packet can only be forwarded to a 1-hop distance in each operational cycle. The improved S-MAC with adaptive listening [4] can improve latency by delivering packets up to a 2-hop distance in each cycle. But the latency is still significant and the use of adaptive listening can significantly increase energy consumption.

Several protocols have been proposed to mitigate packet delivery latency without sacrificing energy efficiency of duty-cycling (e.g., RMAC [5], T-MAC [6] and DW-MAC [7]). Take RMAC for example. Similar to S-MAC, RMAC divides the operational cycle of a sensor node into three periods: *SYNC*, *DATA* and *SLEEP*. But the difference lies in the fact that RMAC delivers a pioneer frame (PION) over multiple hops during the *DATA* period to set up a multi-hop schedule for subsequent data forwarding during the *SLEEP* period. Therefore, this approach can forward a data packet over several hops within a single cycle, thus improving delivery latency. Note that the PION frame has a dual function, one is to request communication like RTS, and the other is to confirm a request like CTS. This PION relaying process continues until either the PION frame has reached the final destination or the current *DATA* period has ended.

Although the improved duty-cycling mechanisms mitigate the delivery latency problem, they were designed independently without considering routing. For example, RMAC assumes a routing protocol has been deployed over it to provide the routing information it needs. But the application of a routing protocol would cause significant performance degradation of these MAC protocols. This paper designs and evaluates a new cross-layer duty cycle MAC protocol with data forwarding supporting a pipeline feature (P-MAC).

P-MAC divides the whole network into several groups around the sink, each with different grades. At the network layer, each sensor node identifies its grade according to its logical hop distance to the sink and simultaneously establishes a sleep/wakeup schedule based on its grade information. The sink is in grade zero and the lower a node’s grade, the fewer hops it needs to send packets to the sink. The wakeup period of a node is divided into two parts, the first one is used for receiving data from the upper grade node, and the second one is used for sending the received data to the lower grade node. Those nodes in the same grade keep the same schedule, which is staggered with the schedule of the nodes in the adjacent grade. Then at the MAC layer, a variation of the RTS/CTS handshake mechanism is used to forward data continuously from the higher grade to lower grade nodes and finally to the sink.

P-MAC does not need an extra independent routing mechanism to support it, thus the communication overhead in the network can be reduced considerably without increasing delivery latency and sacrificing energy efficiency. In addition, each node only maintains a grade and a schedule, which improves the scalability with respect to network topology changes such as nodes dying over time, the later addition of new nodes or nodes moving to different locations. And multiple sinks can be used to partition a large-scale WSN into several independent sub-networks to increase the network manageability and balance energy dissipation.

The remainder of the paper is organized as follows. In Section 2, we discuss the related work in the area of duty cycle MAC designs for WSNs. In Section 3, the details of P-MAC are presented, including the network scalability discussion. Section 4 gives the performance evaluation and analysis based on simulation using the OPNET modeler. Finally, Section 5 concludes the paper.

## 2. Related Work

The original duty cycle MAC protocols for WSNs (e.g., S-MAC [3,4]) introduce significant end-to-end delivery latency. Many researchers have proposed other scheduled MAC schemes to mitigate this problem without sacrificing the energy-efficiency of the duty-cycling mechanism. These approaches can be approximately divided into two categories: synchronous and asynchronous, according to their synchronization requirements. Synchronous schemes (e.g., S-MAC [3], T-MAC [6], RMAC [5] and DW-MAC [7]) require synchronization among neighboring nodes, ensuring that they can cooperate for communication, whereas asynchronous schemes (e.g., B-MAC [8], WiseMAC [9], X-MAC [10] and RI-MAC [11]) allow each node to establish and maintain its own schedule independently, usually using preamble schemes.

T-MAC [6] follows S-MAC using synchronization and virtual clustering schemes. It dynamically ends the active part of the listen/sleep duty cycle to further save energy when there are no packets to receive. However, the higher delivery latency problem still exists in T-MAC, since a data packet can only be forwarded a 1-hop distance within a single cycle. After becoming aware of this latency problem, the designers of S-MAC mitigated the problem by introducing the adaptive listening technique [4]. In the improved S-MAC with adaptive listening, if a node overhears an RTS or CTS, it won't go to sleep when the *SLEEP* period begins but instead will keep awake for a short time. Thereby, if this node is the next-hop node, it can immediately receive data from its neighbor instead of waiting for the next cycle. Thus, a packet can be delivered up to a 2-hop distance within a single cycle. However, the improvement is minor and this technique also consumes more energy, because many neighboring nodes need to keep awake during adaptive listening, but only one of them will be the next hop.

RMAC uses a PION frame to reserve a channel over several hops during the *DATA* period, then, it transmits a data packet through the reserved channel during the *SLEEP* period. Therefore, the data packet can be forwarded across multiple hops within a single cycle, which not only reduces delivery latency significantly but also handles traffic contention efficiently. The recently proposed PRMAC [12] inherits this advantage and exploits it further by using PION to schedule multi-hop transmission of multiple data packets, enabling multiple packets to be transmitted over multiple hops within a single cycle, thus ensuring that PRMAC can respond to traffic load changes better than RMAC.

Unlike the above synchronous duty cycle MAC protocols, asynchronous schemes [8–10,13] using the preamble sampling technique [14] were introduced into the MAC layer design for WSNs. The basic idea of this strategy is that prior to data transmission, a sender transmits a long enough preamble lasting at least as long as the receiver's sleep period. The receiver periodically wakes up and checks for activity on the channel. If a preamble is detected, the receiver keeps awake long enough to receive the data, otherwise it goes back to sleep. RI-MAC [11] differs from this original asynchronous strategy since it uses the receiver-initiated mechanism, which is similar to that proposed in [15] for general wireless networks, to achieve better performance, in which the sender keeps active until the receiver explicitly informs the sender when to start data transmission by sending a short beacon frame.

Hybrid approaches with channel polling scheme, such as SCP [16] and LEMR [17] have also been proposed. Compared with these duty cycle MAC protocols, P-MAC fully integrates routing into a wake-up scheduling algorithm. In P-MAC, the whole network is divided into several grades, which are used to guide data transmission. The schedules between two adjacent grades are staggered so that data can be transmitted continuously to the sink in a pipeline fashion, thus ensuring that the delivery latency is more acceptable. Actually, this pipeline scheduled pattern scheme for reducing latency is not original. For example, DMAC [18] allows continuous packet forwarding by offsetting a sensor node’s sleep schedule (like a pipeline) based on a tree communication structure. Li *et al.* [19] and Cao *et al.* [20] also proposed a similar pipelining scheme. Keshavarzian *et al.* [21] evaluated several existing scheduling schemes including a staggered ladder pattern scheme. P-MAC combines this scheduling scheme with grade division for routing to achieve high energy efficiency and low delivery latency.

## 3. P-MAC Design Integrated with Routing

The conventional duty cycle MAC protocols were designed independently neglecting the impact of the network layer. P-MAC considers cross-layer optimization with the goal of minimizing the communication overhead and maintaining the superiority of duty-cycling schemes. It divides all sensor nodes into different grades according to their logical hop distances to the sink. The lower a node’s grade, the fewer hops it needs to send packets to the sink.

### 3.1. Network Model

P-MAC is proposed for WSNs deployed for rare events detection with prompt reporting. Applications including fire or other hazards detection fall into this category. Such a network consists of many sensor nodes randomly deployed in a sensing area with one (or a few) sink(s) collecting information for an outside system. All nodes are assumed to be homogeneous with the same transmission range.

### 3.2. Grade Division and Schedule Assignment (GDSA)

Before starting data transmission, each node need to find a grade it belongs to, and choose a schedule for periodically listening and sleeping. This is implemented by operating the *GDSA* mechanism at the network layer of each node.

GDSA is initiated from the sink. In *GDSA*, each node maintains grade information (denoted by *G_n_*) with an initial value of −1, except that the sink’s grade is zero (*G_n_* = 0) at all times. The sink first chooses a schedule according to its grade (the schedule choosing rule will be introduced in detail in Section 3.5). Then it generates a GRADE message containing a field denoted by *G_m_*. After setting *G_m_* to one, the sink broadcasts this message. A node receiving a GRADE message with *G_m_* = *i* sets its grade to *i* (*G_n_* = *i*), chooses a schedule corresponding with its grade, and rebroadcasts the message after increasing *G_m_* by one, unless it has already joined an equal or a lower grade. The pseudo-code of the algorithm used by a node for processing the received GRADE message is shown in Algorithm 1.

**Figure 1.**
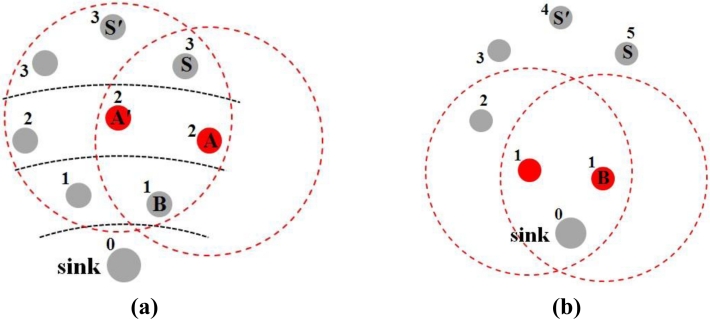
**(a)** After grade division, the network is divided into several annular grades similar to concentric circles centered at the sink; **(b)** if *A′* and *A* do not exist, *S′* and *S* are in grade 4 and 5, respectively.

After the grade division using the above scheme, the whole network is divided into several annular grades similar to concentric circles with the center at the sink, as shown in Figure 1(a). But this is not absolute. For example, if node *A* and *A′* do not exist, the network may be divided into the formation shown in Figure 1(b).

As stated above, each node simultaneously establishes a periodical sleep/wakeup schedule according to its grade information during the grade division process. Those nodes in the same grade keep the same schedule, but schedules are staggered between two adjacent grade nodes. Suppose a node is in grade *i* (*i* > 0), it repeatedly experiences three periods: receiving data from the (*i*+1)th grade node (the *RECEIVE DATA* period), sending data to the (*i*-1)th grade node (the *SEND DATA* period), and sleeping (the *SLEEP* period). If the upper grade nodes are in the *SEND DATA* period, their adjacent lower grade nodes must be in the *RECEIVE DATA* period (the duration of each period will be analyzed in Section 3.4). Thereby, data frames can be forwarded continuously in a pipeline way from the source node to the sink, thus making the delivery latency acceptable. Those adjacent nodes in the same grade contend with each other for the shared medium while those in different grades cooperate with each other for data transmission.

The pipelining concept cited in this paper is defined as follows:

*Definition 1 (Pipelining):* In duty-cycling MAC protocols, a node generally can complete receiving a data frame from one of its upstream nodes and then sending this data frame to one of its downstream nodes within one cycle. This is called pipeline data transmission.

### 3.3. Data Transmission

After completing *GDSA*, a sensor node with pending data will not be aware of any concrete routing paths to the sink, but it can use a variation of the RTS/CTS handshake mechanism at the MAC layer to determine the next-hop node from its adjacent lower grade nodes. There are two differences between the variation and the original RTS/CTS handshake in IEEE 802.11. First, in P-MAC, the RTS sent by a source node contains the node’s grade information passed down from the network layer instead of a concrete next-hop address. All nodes in the adjacent lower grade can reply to the source node with CTS if they receive the RTS. Second, the Contention Window (CW) is also used when a node replies with CTS in P-MAC, because several lower grade nodes will simultaneously receive the RTS, and there will be contention for data relaying among these nodes.

**Figure 2.**
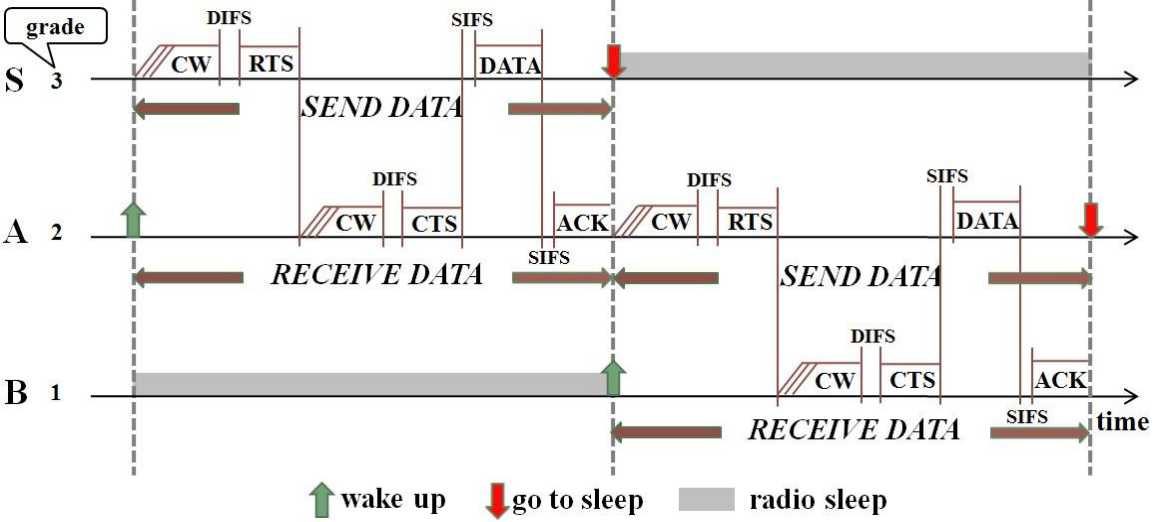
P-MAC integrated with routing. Node *S* has data to send to the sink.

Consider the network shown in Figure 1(a). When nodes in grade 3 are in the *SEND DATA* period, those nodes in grade 2 are in the *RECEIVE DATA* period. Suppose node *S* has data to send to the sink. *S* first broadcasts the RTS frame in its *SEND DATA* period after contending with its neighboring nodes in the same grade (e.g., *S′*) and winning the medium. Both node *A* and *A′* can receive this RTS in their *RECEIVE DATA* periods. They contend with each other for replying with CTS. If node *A*’s CTS is first received by node *S*, *S* will send its data to *A*. After *A* receives the data, it sends an ACK frame to *S*. Then *A* waits to enter its *SEND DATA* period. After receiving the ACK, *S* enters sleep mode. Those nodes (e.g., *S′* and *A′*) that failed in the channel contention will go to sleep and wait to enter their subsequent periods. In the same manner, node *A* finds its next relaying node *B* and sends data to *B* in its *SEND DATA* period. Figure 2 shows this data transmission process.

Note that the Network Allocation Vector (NAV) in IEEE 802.11-style MAC protocols for virtual carrier sense is not used in P-MAC, because each node in P-MAC only receives data during its *RECEIVE DATA* period and only sends data during its *SEND DATA* period, respectively. If a node fails in the contention for receiving/sending data or it has waited a long enough time without receiving RTS/CTS, the node will go to sleep. Thus, the virtual carrier sense used for collision avoidance is not necessary in P-MAC.

### 3.4. Duration of Each Period

An analysis on the duration of each period in the P-MAC cycle follows:

(1) SEND/RECEIVE DATA period: As shown in Figure 2, the duration of the *SEND DATA* period in node *S* is identical to the duration of the *RECEIVE DATA* period in node *A*, if node *S* and *A* are involved in the current data transmission. The maximum time of the *SEND/RECEIVE DATA* period, *T_S/R_*, is calculated as follows:

(1) $$T_{S/R}=2\text{CM}+2\text{DIFS}+2\text{SIFS}+\text{durRTS}+\text{durCTS}+\text{durDATA}+\text{durACK},$$

where *durRTS*, *durCTS*, *durDATA* and *durACK* are the transmission duration of RTS, CTS, DATA and ACK, respectively.

(2) SLEEP period: In P-MAC, the duration of the *SLEEP* period is determined by the following equation:

(2) $$T_{\text{SLEEP}}=\text{sleep}\_\text{factor}\cdot T_{S/R},$$

where *sleep_factor* is a positive integer. The whole cycle duration is given by:

(3) $$T_{\text{cycle}}=(\text{sleep}\_\text{factor}+2)\cdot T_{S/R}=\tau \cdot T_{S/R},$$

where *τ* is called *cycle coefficient*. Next we discuss how to determine the value of *sleep_factor*.

**Figure 3.**
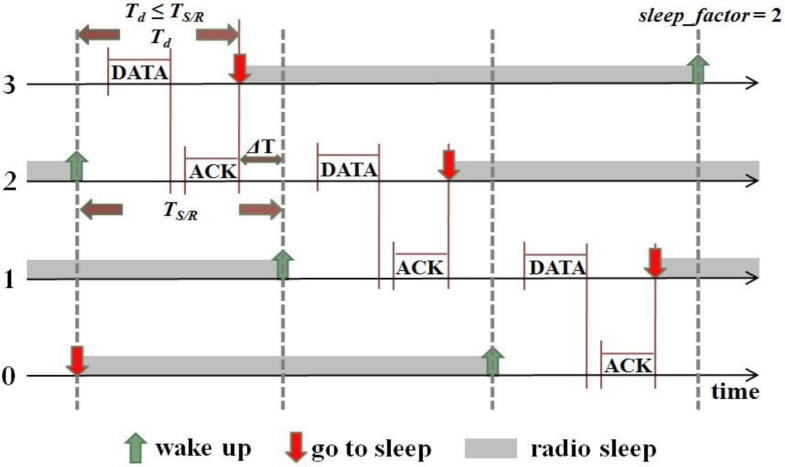
An example illustration of *sleep_factor*, data transmission route: 3**→**2**→**1**→**0.

Figure 3 shows an example used for analysis. For simplicity, the RTS/CTS handshake process is not shown. There are four nodes, where node 0’s grade is zero (it is the sink node), node 1’s grade is one, and so on. Node 3 has data to send to node 0. *T_d_* is the actual duration time for a node to finish its one-hop data transmission process. Note that *T_d_* ≤ *T_S/R_*, since the node may not select the last slot in CW. Thus the node’s actual sleep time is *ΔT + T_SLEEP_*, where *ΔT* = *T_S/R_* – *T_d_*. Anyway, each node can sleep for at least *T_SLEEP_* time in each cycle.

If *sleep_factor* is a positive integer, for example, *sleep_factor* = 1, when the *i*th (*i* > 0) grade nodes wake up, the (*i* – *1*)th grade nodes are still in the *SLEEP* period, and the (*i + 2*)th grade nodes will go to sleep for at least *sleep_factor* · *T_S/R_* = *T_S/R_* time. Therefore, the communications between the *i*th grade nodes and the (*i* + 1)th grade nodes will not be interfered with by the (*i* – 1)th and (*i* + 2)th grade nodes. Considering that the interference range is about two times the transmission range, the value of *sleep_factor* needs to be at least 2. Figure 3 shows the case of *sleep_factor* = 2.

### 3.5. Schedule Choosing Rule and Synchronization

Suppose the time needed to finish the *GDSA* process is within *T_g_* time. The transmission duration of a GRADE message is denoted by *durGRADE*. Once a node receives a GRADE message and updates its grade to *i*, it uses the following rules to choose an initial schedule:

1. if *i* % *τ*= 0, the node will enter the *RECEIVE DATA* period after (*T_g_* – *i* · *durGRADE*) time.
2. if *i* % *τ*= 1, the node will enter the *SEND DATA* period after (*T_g_* – *i* · *durGRADE*) time.
3. if *i* % *τ* ≥ 2, the node will enter the *SLEEP* period after (*T_g_* – *i* · *durGRADE*) time. And its sleep duration should be:
  (4) $$\text{time}=T_{\text{SLEEP}}-(i\%\tau -2)\cdot T_{S/R}=(\tau -i\%\tau )\cdot T_{S/R}$$

Note that *T_g_* should be long enough to enable each node in the network to get its grade and corresponding schedule after the *GDSA* process.

Similar to S-MAC [3] and RMAC [5], synchronization is also required among neighboring nodes in P-MAC to solve the problem of clock drift as time advances. However, the difference is that P-MAC doesn’t use an extra period (e.g., *SYNC*) to achieve this goal. In P-MAC, each frame contains the relative start time of the current period. When a sensor node receives a frame, it will adjust its schedule if the clock drift is too serious. If a node fails to receive correct data for a long enough time, it will stay active and listen to the channel for some time to revise its grade and/or schedule. This loose synchronization mechanism is used because in P-MAC, a node with pending data to send may have several adjacent lower grade nodes, any of which may become the relay node. We suppose this mechanism will guarantee reliability for data forwarding.

### 3.6. Scalability Discussion

**Figure 4.**
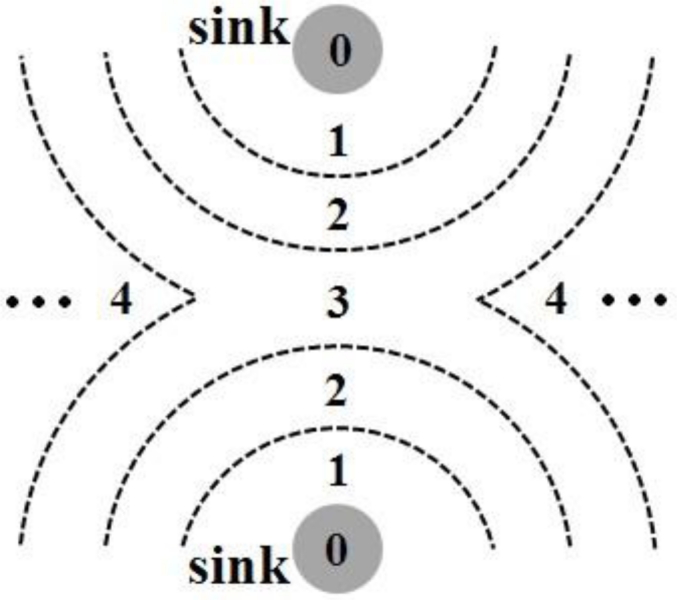
Two-sink case. Each number denotes a corresponding grade.

P-MAC is a cross-layer duty cycle MAC protocol seamlessly integrated with routing function. It not only reduces the protocol overhead, but also helps enhance scalability when there are changes in network topology:

1. If a new node is added to the network, it will identify which grade it should join and the corresponding schedule it should choose after listening to its neighbors for some time. Subsequently, it can join the network for data transmission.
2. It is relatively easy for P-MAC to support node mobility. A mobile node needs to redefine its grade and schedule if it moves out of its original grade area. Like a newly added node, it can rejoin the network later, unless it moves out the network and thus becomes isolated.
3. In a large-scale WSN, multiple sinks are needed to increase the network manageability and balance energy dissipation. P-MAC can partition the whole network into several sub-networks using its grade division mechanism. Each sink serves one sub-network independently. Figure 4 shows a two-sink case. For a data-gathering network, all sinks are expected to be connected to an outside system. Hence, it is irrelevant which exact sink receives the data information.

No further elaboration on these issues is presented in this paper, but we believe these issues are reasonable and provide a guideline for future exploration.

## 4. Simulation Evaluation

### 4.1. Simulation Parameters

**Figure 5.**
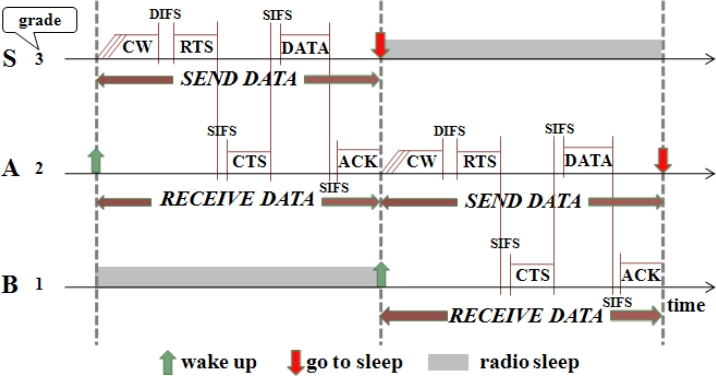
Basic P-MAC without routing function.

In this section, we evaluate the P-MAC design in comparison with S-MAC and RMAC using the OPNET modeler. For fairness, we give the simulation result of both the basic P-MAC without the routing function and the full P-MAC that is seamlessly integrated with routing. In comparison with the full P-MAC, the difference is that in the basic P-MAC, the RTS is sent to a concrete next node, which has its address passed down by the networking layer. So only the node for which the RTS is destined will reply with CTS without waiting for CW time. Figure 5 illustrates the basic P-MAC, and the maximum time of the *SEND/RECEIVE DATA* period, *T_S/R_*, is shown below. This result differs from that calculated by Equation (1):

(5) $$T_{S/R}=\text{CW}+\text{DIFS}+3\text{SIFS}+\text{durRTS}+\text{durCTS}+\text{durDATA}+\text{durACK}.$$

Each node uses the two-ray ground radio propagation model and has a single omni-directional antenna. Table 1 shows the key networking parameters used in our simulation. These parameters are the default settings in the standard S-MAC simulation module distributed with the ns-2.29 package. The sizes and transmission latencies of different types of packets are shown in Table 2, and the settings are the same as in [5].

PION relaying number *N* in RMAC defines the distance (hops) a PION frame can be forwarded in the *DATA* period. In our simulation, we set *N* = 4, as in [5]. The cycle related parameters are shown in Table 3. Both S-MAC and RMAC keep the same duty cycle (about 6%). Because the cycle division in P-MAC is different from that of both S-MAC and RMAC, the full P-MAC is set with *sleep_factor* = 14 to have the same cycle duration as RMAC and the basic P-MAC is set with *sleep_factor* = 21 to have a similar cycle duration to the full P-MAC.

### 4.2. Simulation Topology

**Figure 6.**
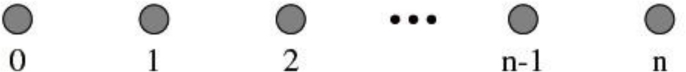
Chain topology.

We use two types of topologies in our simulations: chain topology and random topology. Figure 6 shows the chain topology. All nodes are equally spaced in a straight line with a 200 meter interval between neighboring nodes. Node 0 sends packets to node *n* through a single CBR (constant bit rate) flow. The hop length of the chains varies from 1–24 hops. For S-MAC, RMAC and the basic P-MAC, the routes for data transmission are assigned manually.

**Figure 7.**
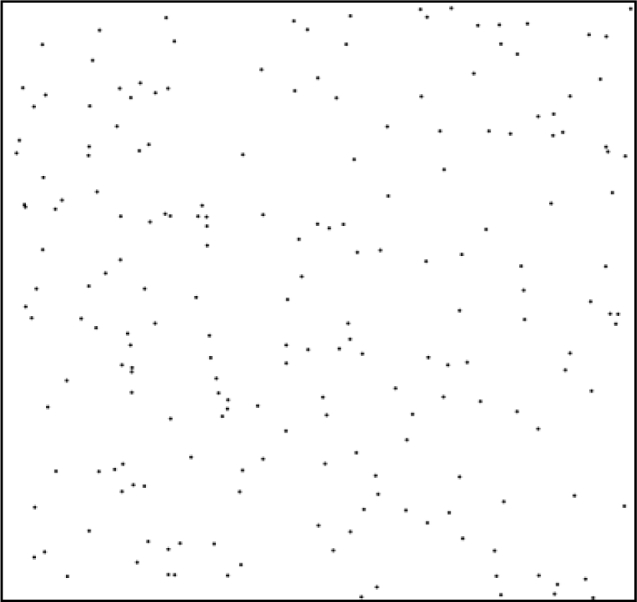
Random topology with 200 sensor nodes in 2,000 × 2,000 m^2^ area.

Figure 7 shows an example of the random topology, which consists of 200 sensor nodes and a sink node (not shown in the figure). The 200 sensor nodes are randomly distributed in a 2,000 × 2,000 m^2^ square area, and the sink node is located at the top-left corner of the square.

The full P-MAC integrated with routing needn’t consider the routing issue. For fairness, we propose a routing mechanism for S-MAC, RMAC and the basic P-MAC by modifying the *GDSA* process in P-MAC, since it is not convenient to assign routes manually in the random topology. In the modified *GDSA* process, each node maintains a routing table containing only one field to record its next-hop nodes’ IDs. The process has the following steps:

1. The sink node with its grade set to zero initiates this process by generating a GRADE message packet. The packet contains two fields denoted by *G_m_* and *ID_ph_*. After setting *ID_ph_* to its own ID and *G_m_* to one, the sink node broadcasts this message.
2. Upon receiving a GRADE message, each node decides whether to update its routing table and/or rebroadcast the message, as shown in Algorithm 2.

**Figure 8.**
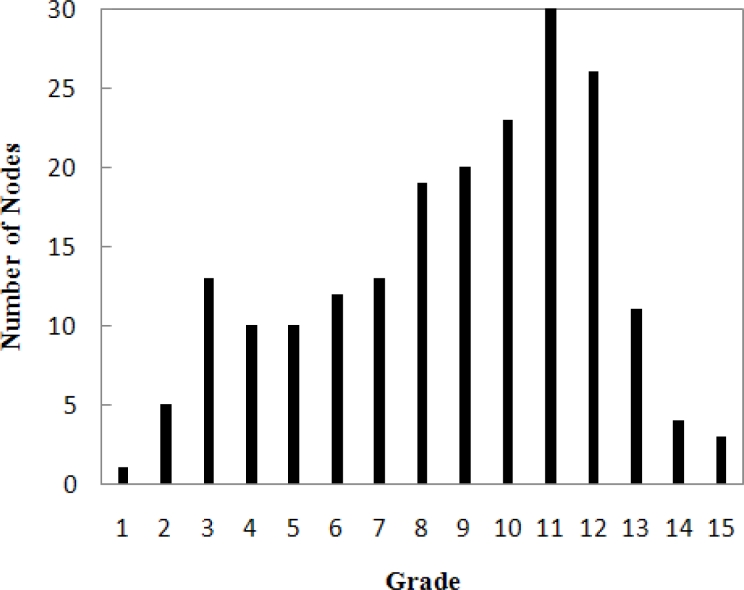
The number of nodes in each grade in the random topology network.

The completion of this process will result in one or more entries in the routing table of each node. During data transmission, the current node randomly chooses one entry and extracts the ID from the entry as the next-hop ID. Also, each node maintains a grade representing the path length from it to the sink. Figure 8 shows the number of nodes in each grade.

In addition, we make the following assumptions. For S-MAC and RMAC, all nodes have already been synchronized to use a single schedule. There is no synchronization traffic during the simulation, but the SYNC period is still contained in the cycle. The original or modified GDSA process is executed only once during the initial phase and the corresponding overhead is not considered during the comparison among S-MAC, RMAC and P-MAC.

### 4.3. Simulation Result

We first evaluate the performance of the end-to-end delivery latency. For the chain topology, node 0 generates a CBR flow at the rate of 1 packet every 10 seconds. The simulation time is set as 1,200 seconds. For the random topology, a random sensor node is selected every 10 seconds to send one packet to the sink. The simulation time is set as 19,200 seconds.

**Figure 9.**
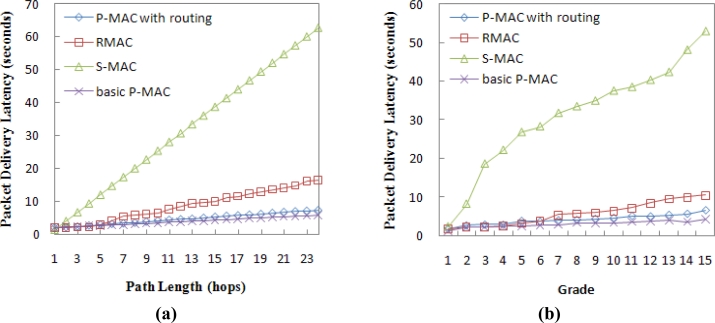
**(a)** Delivery latency in the chain topology. **(b)** Delivery latency in random topology.

Figure 9 shows the growth trend of the average packet delivery latency with respect to the increase in path length in the chain topology and random topology, respectively. Both figures show that the delivery latency in P-MAC increases at a much lower rate than S-MAC and RMAC. This is because S-MAC only forwards a packet to a 1-hop distance in each operational cycle and RAMC has to wait for the start of the next *DATA* period to forward data again if the path length exceeds the PION relaying number, while P-MAC can forward data in a pipeline fashion.

Provided the path length does not exceed the PION relaying number, data transmission can be completed within a single cycle in RMAC, spending about *T_DATA_* time. For P-MAC, the data frame moves to a 1-hop distance per *SEND DATA* period, spending about *T_S/R_* time. In our simulation, *T_DATA_* (168.0 ms) is 66.0 ms less than *T_S/R_* (234.0 ms), so Figure 9 shows that RMAC has slightly less delivery latency when the path length is within 4 hops, as *N* = 4 in our simulation.

Figure 9 also shows that the basic P-MAC has lower delivery latency than the full P-MAC with routing, since the RTS is sent to a certain node during data transmission in the basic P-MAC, which reduces network congestion and improves the data delivery ratio.

Next, we evaluate the network throughput and energy efficiency for P-MAC. The network throughput is recorded in terms of the average number of packets successfully received by the sink per second. For the chain topology, we keep *n* = 24, and the data input interval for node 0 varies from 10–1 seconds. For the random topology, the interval for randomly selecting a sensor node to send data varies from 10-1 seconds. In both topologies, the simulation time is set as 1,200 seconds.

**Figure 10.**
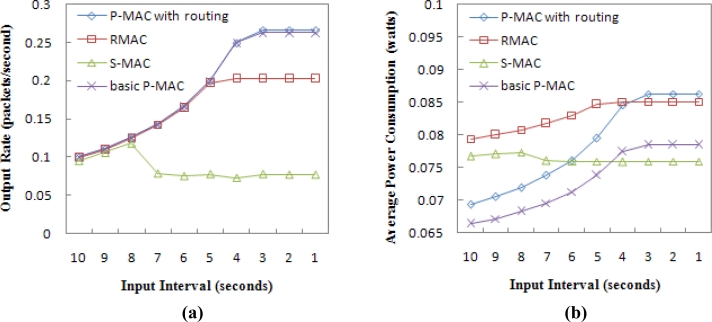
**(a)** Throughput in the chain topology. **(b)** Average power consumption per sensor node in the chain topology.

In the chain topology, Figure 10(a) shows that when the input interval is less than 8 seconds, the output rate in S-MAC decreases rapidly, since S-MAC has poor traffic contention handling due to its weakness of forwarding a packet to a 1-hop distance in each operational cycle. Because the network throughput of S-MAC decreases when the input interval is less than 8 seconds, the corresponding energy consumption also decreases, as shown in Figure 10(b).

For P-MAC and RMAC, Figure 10(a) shows that the output rate follows the input rate until the input interval is less than 5 seconds, and finally reaches the steady state. When the network throughput reaches its peak point, the incoming injected packets cannot continue to be sent, and the network energy consumption will not be increased, as shown in Figure 10(b). When the input interval is smaller than 4 seconds, P-MAC with routing will have higher energy consumption than RMAC because of its higher throughput. The basic P-MAC has almost the same throughput as the full P-AMC but has lower energy consumption, since it doesn’t consider the routing issue.

**Figure 11.**
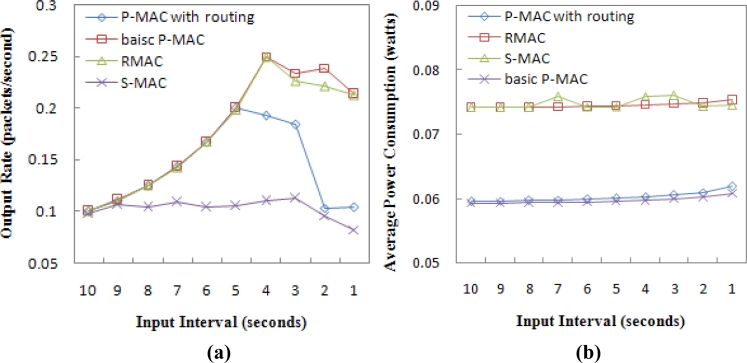
**(a)** Throughput in the random topology. **(b)** Average power consumption per sensor node in the random topology.

In the random topology, Figure 11(a) shows that RMAC has better performance than the P-MAC integrated with routing in terms of throughput. But the basic P-MAC has better throughput than RMAC when the input interval is less than 4 seconds. Figure 11(b) shows that P-MAC is more energy efficient than RMAC and S-MAC in the random topology. For all protocols, the range of improvement of the average power consumption is not obvious as the input interval decreases. This is because each point on the curve represents the average of 200 nodes, many of which did not participate in packet relaying as much as the nodes in the chain topology.

Finally, we evaluate how the variation of *sleep_factor* in P-MAC integrated with routing affects the network performance. The value of *sleep_factor* determines the length of the *SLEEP* period (see Equation 2). Increasing *sleep_factor* increases the length of the *SLEEP* period time and the whole cycle time. Increasing the sleeping time of sensor nodes can save more energy. However, when a packet is not generated at the start of the *SEND DATA* period in the current cycle, a longer cycle time makes the packet wait longer for the next cycle.

**Figure 12.**
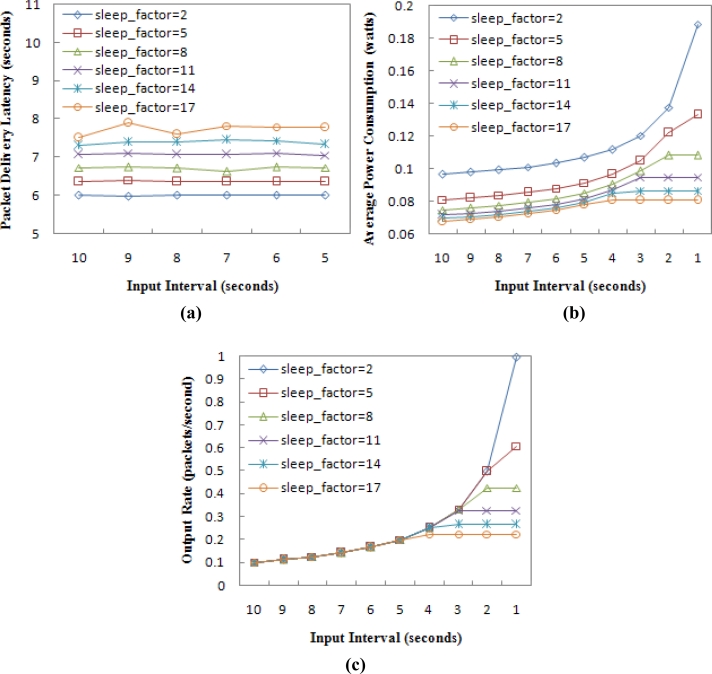
The impact evaluation of *sleep_factor* in the chain topology, **(a)** Packet delivery latency. **(b)** Average power consumption. **(c)** Output rate.

For the chain topology, it is still the case that *n* = 24. The input interval of a CBR flow in node 0 varies from 10–1 seconds. Each simulation runs for 1,200 seconds. Table 4 shows the different values of *sleep_factor* we used in our simulation, as well as their corresponding *SLEEP* period time and cycle time. The average packet delivery latency, the average power consumption per sensor node and the data throughput are observed, and the simulation results are shown in Figure 12.

Note that Figure 12(a) shows the delivery latency that only varies with respect to the input interval from 10–5 seconds. This is because when the input interval is less than 5 seconds, the latency in the network with *sleep_factor* = 17 will be too large to be displayed. Figure 12(a) implies that the packet delivery latency increases as the *sleep_factor* increases for the reason stated above. But a greater *sleep_factor* ensures that nodes save more energy, as shown in Figure 12(b). When the network throughput is considered, a small *sleep_factor* is expected if the network traffic load is high, as shown in Figure 12(c). Figure 12(c) also shows that the cycle time is almost equal to the lowest input interval, which can still ensure that the output rate follows the input rate. For example, when *sleep_factor* = 17, the cycle duration is about 5 seconds, which is the lowest input interval ensuring that the network has 100% throughput, as shown in Figure 12(c).

**Figure 13.**
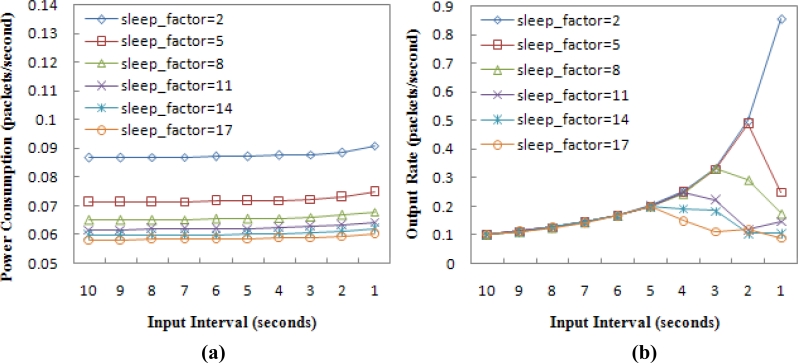
The impact evaluation of *sleep_factor* in the random topology, **(a)** Average power consumption. **(b)** Output rate.

Figure 13 shows the impact evaluation of *sleep_factor* in the random topology. The result is in accord with that in the chain topology. Note the evaluation doesn’t include a delivery latency evaluation, which is not convenient to be given for the random topology. Designers can select the value of *sleep_factor* according to the network traffic load. For example, if the network input rate doesn’t exceed 0.2 packets/second (the input interval is not less than 5 seconds), *sleep_factor* = 17 should be chosen, since the throughput can reach 100% with the lowest energy consumption. But if the input rate reaches 1 packet/second or higher, the minimum value of *sleep_factor* = 2 should be chosen.

## 5. Conclusions

Conventional duty cycle MAC protocols are energy-efficient and some of them also have mitigated other existing problems such as the delivery latency problem, but they have been designed independently without considering routing. Adding a routing protocol would cause significant performance degradation of the whole network. The P-MAC design presented in this paper is a cross-layer duty cycle MAC protocol seamlessly integrated with routing function. It uses the *Grade Division and Schedule Assignment* (*GDSA*) scheme at the network layer to assign all sensor nodes into different grades around the sink and ensures that nodes maintain staggered schedules between any two adjacent grades. Then a variation of the RTS/CTS handshake mechanism is used at the MAC layer to forward data continuously in a pipeline fashion from the higher grade to lower grade nodes and finally to the sink. The communication overhead in the network can be significantly reduced while maintaining the superiority of duty-cycling schemes. The simulation evaluations show that P-MAC achieves better performance in terms of energy efficiency, latency reduction and throughput improvement. Future research needs to study network scalability, which was not adequately addressed.

## Figures and Tables

**Table 1.** Networking Parameters.

**Bandwidth**	20 Kbps	**Tx Range**	250 m
**Idle Power**	0.45 W	**Carrier Sensing Range**	550 m
**Sleep Power**	0.05 W	**Contention Window (CW)**	64 ms
**Rx Power**	0.5 W	**DIFS**	10 ms
**Tx Power**	0.5 W	**SIFS**	5 ms

**Table 2.** Transmission Duration Parameters.

	**Frame Size (bytes)**	**Tx Latency (ms)**

**RTS/CTS**	10	11.0
**ACK**	10	11.0
**PION (RMAC)**	14	14.2
**DATA**	50	43.0

**Table 3.** Cycle Duration Parameters.

	***T****_SYNC_*	***T****_DATA_*	***T****_SLEEP_*	***T**_cycle_*
**RMAC**	55.2 ms	168.0 ms	3,520.8 ms	3,744.0 ms
**S-MAC**	55.2 ms	104.0 ms	2,511.2 ms	2,670.4 ms
	***T****_S/R_*	***sleep_factor***	***T****_SLEEP_*	***T****_cycle_*
**Basic P-MAC**	234.0 ms	21	3,465.0 ms	3,795.0 ms
**Full P-MAC**	234.0 ms	14	3,276.0 ms	3,744.0 ms

**Table 4.** Cycle duration with different *sleep_factor*.

***sleep_factor***	***T****_S/R_***(ms)**	***T****_SLEEP_***(ms)**	***T****_cycle_***(ms)**

2	234	468	936
5	234	1,170	1,638
8	234	1,872	2,340
11	234	2,574	3,042
14	234	3,276	3,744
17	234	3,987	4,446

**Algorithm 1.** *GDSA*: processing the received GRADE message.

1:	**if***G_n_* < 0 **then**
2:	*G_n_***←***G_m_*
3:	choose a corresponding schedule
4:	*G_m_***←***G_m_* + 1
5:	rebroadcast the GRADE message
6:	**else if***G_n_* > *G_m_***then**
7:	*G_n_***←***G_m_*
8:	update the node’s schedule
9:	*G_m_***←***G_m_* + 1
10:	rebroadcast the GRADE message
11:	**else**
12:	discard the GRADE message
13:	**end if**

**Algorithm 2.** The modified *GDSA*: processing the received GRADE message.

1:	**if***G_n_* < 0 || *G_m_* < *G_n_***then**
2:	**if***G_m_* < *G_n_***then**
3:	clear the current node’s routing table
4:	**end if**
5:	*G_n_***←***G_m_*
6:	add *ID_ph_* into the current node’s routing table
7:	*G_m_***←***G_m_* + 1
8:	*ID_ph_***←** the current node’s ID
9:	rebroadcast the GRADE message
10:	**else if***G_n_* == *G_m_***then**
11:	add *ID_ph_* into the current node’s routing table
12:	discard the GRADE message
13:	**else**
14:	discard the GRADE message
15:	**end if**
